# Change of Pathological Type to Metaplastic Squamous Cell Carcinoma of the Breast During Disease Recurrence: Case Report and Literature Review

**DOI:** 10.3389/fonc.2020.00032

**Published:** 2020-02-25

**Authors:** Tianhui Guo, Zhiying Chen, Jinpeng Xu, Yongchun Zhang

**Affiliations:** ^1^Department of Radiation Oncology, Laoshan Branch of Affiliated Hospital of Qingdao University, Qingdao, China; ^2^Department of Radiation Oncology, The Affiliated Hospital of Qingdao University, Qingdao, China

**Keywords:** breast cancer, recurrence, metaplastic squamous cell carcinoma, treatment, case report

## Abstract

**Background:** Metaplastic squamous cell carcinoma (SCC) of the breast is a rare and heterogeneous group of primary breast malignancies. The etiology, pathogenesis, and proper treatment for this kind rare breast cancer are still unclear.

**Case presentation:** We reported a case of a 55-year-old woman with a palpable lump in the inner quadrant of the right breast. She underwent a right breast mass resection and sentinel lymph node biopsy, which revealed that the tumor was an invasive ductal carcinoma, followed by four cycles of doxorubicin plus cyclophosphamide and four cycles of docetaxel as adjuvant chemotherapy, and then simultaneous integrated boost intensity modulated radiotherapy to the whole right breast. After 2 years' follow-up, she had biopsy-proven disease recurrence in the right breast, which revealed SCC, and a mammogram showed abnormalities in the lower inner quadrant of the right breast and left axillary lymph nodes. Then we performed bilateral breast modified radical mastectomy, which confirmed that the recurrent tumors were metaplastic SCC, followed by adjuvant chemotherapy and adjuvant radiotherapy of the left supraclavicular and apical axillary regions. There has been no recurrent or metastatic evidence in the 16 months' follow-up since the second surgery.

**Conclusion:** This case report shows that evolution of pathology type in recurrent breast cancer after initial treatment is possible. Detailed pathologic and immunohistochemical analyses are needed for identification of this change. Surgery and adjuvant radiation and chemotherapy are appropriate treatments for recurrent primary SCC of the breast.

## Background

Breast cancer is the most common cancer of women in China and the United States, for which the clinical manifestation and the biological behavior are heterogeneous (1, 2). The most common pathological types of the breast cancer are ductal carcinoma and lobular carcinoma, which account for more than 70% of breast carcinoma. Metaplastic breast carcinoma, however, is a rare and heterogeneous group of primary breast malignancies accounting for < 1% of invasive breast cancer (3). These tumors including the type of squamous cell carcinoma (SCC) are non-glandular differentiation. The etiology and nosogenesis of metaplastic breast carcinoma are still uncertain. There are several hypotheses for the pathogenesis of breast SCC. One theory is that the lesion is adenocarcinoma with an excessive form of squamous metaplasia (4). Another one is that it is straightly developed from the epithelium of the mammary ducts; moreover, an alternate theory is that the tumor arises from foci of squamous metaplasia within a pre-existing adenocarcinoma of the breast (5).

Because primary breast SCC is rare, a detailed metastatic workup is needed to rule out the possibility of metastatic disease. There are no characteristic clinical and imaging manifestations for SCC of the breast. Hence, the nature of the lesion needs to be determined by the pathology (4). In this paper, we presented a report of recurrent breast cancer of which the pathological type changed from the ductal carcinoma to metaplastic SCC.

## Case Presentation

**Figure 1:**
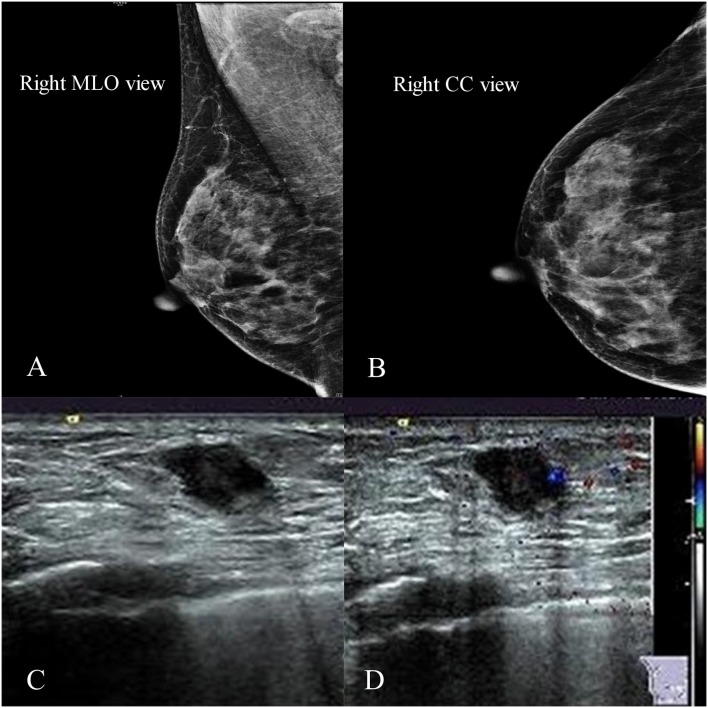
Imaging of the primary tumor. **(A,B)** Right mammogram showed that the local structure of the inner quadrant of the right breast was crowding. **(C,D)** Ultrasound showed an irregular hypoechoic mass of 1.2 × 0.8 cm located at 3–4 o'clock.

In 2015, a 55-year-old woman with a palpable lump in the inner quadrant of the right breast presented to the Breast Center in our hospital. She had no other clinical symptoms such as pain, skin change, nipple retraction, or nipple discharge. She was a non-smoker and denied having any systemic diseases or any family history of breast or ovary cancer, but her father died of gastric adenocarcinoma, and her mother died of lung cancer. On physical examination, there was a 2 × 1 cm mass at 3 o'clock in the right breast, 2 cm away from the nipple. The lump was firm, border unclear, moveable, irregular, and not fixed to the skin or chest wall. No abnormality was found in the axillary or supraclavicular lymph nodes. A mammogram showed a lesion classified as Breast Imaging Reporting and Data System 4B in the inner quadrant of the right breast (Figures 1A,B). The ultrasound showed an irregular hypoechoic mass of 1.2 × 0.8 cm located at 3–4 o'clock, and no positive lymph node was detected (Figures 1C,D).

**Figure 2:**
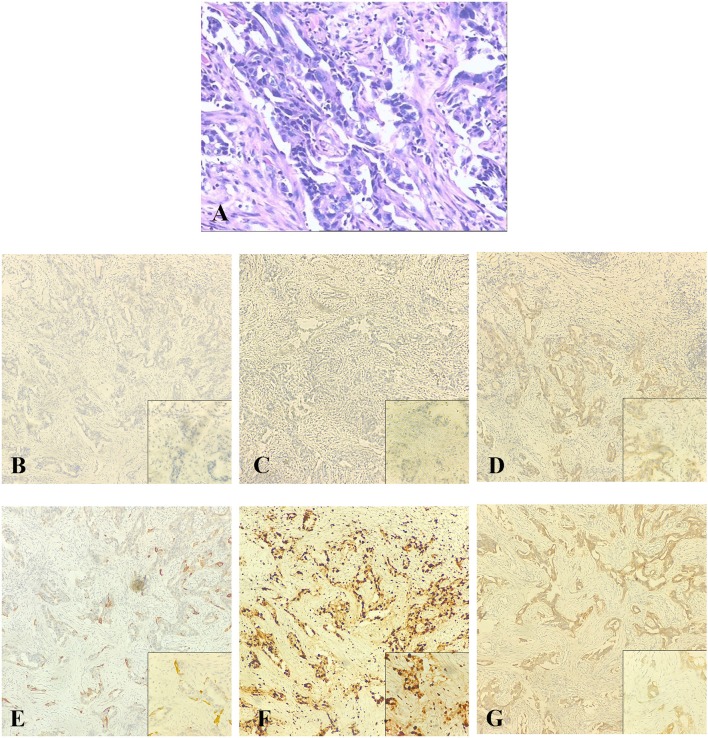
The postoperative pathological examination with hematoxylin and eosin (HE) staining and immunohistochemical (IHC) staining of the primary tumor in 2015. **(A)** Histological findings with HE staining at high magnification (40×) showed that the tumor was an invasive ductal carcinoma. **(B–G)** IHC staining was used to detect the expressions of ER, PR, HER-2, CK5/6, Ki-67, and EGFR at original magnification (10×). **(B)** ER negative. **(C)** PR negative. **(D)** HER-2 weakly positive. **(E)** CK5/6 negative. **(F)** Ki-67 positive (80%). **(G)** EGFR focal positive.

We performed an ultrasound-guided core needle biopsy, which confirmed the diagnosis of invasive ductal carcinoma (IDC). After detailed discussion with the surgeon, the patient chose to preserve her breast. So we performed a right breast mass resection and sentinel lymph node biopsy on December 14, 2015. An invasive carcinoma in the mammary gland was localized in the lower inner quadrant with a maximum diameter of 0.6 cm. Histologically, the tumor was predominantly grade 2 IDC (about 60%), partial invasive micropapillary carcinoma (about 20%), and partial ductal carcinoma *in situ* (about 20%) with focal dysplasia in the upper resection margin (Figure 2). There was no metastasis in the sentinel lymph nodes (0/6). Immunohistochemical staining showed that the tumor was ER-negative, PR-negative, CerbB-2–positive (intensity 1), CK5/6-negative, P53-positive 70%, EGFR-focal positive, and Ki-67–positive 80%, and a FISH test demonstrated negative Her-2 gene amplification (Table 1). Thus, the tumor was pT1N0M0, equivalent to stage Ia.

**Table 1:** The patient's characteristics and treatment process.

**Gender**	**Female**		
Age, year	55		
Process	Primary diagnosis	November, 2015 3 o'clock of the right breast	
	Primary pathology	Predominant invasive ductal carcinoma	
		Immunohisto-chemistry	ER(–), PR(–), HER-2(1+), CK5/6(–), Ki-67(+,80%), EGFR(focal+), E-Cadherin(+)
	Treatments	Surgery	Mass enlargement resection Sentinel lymph node biopsy
		Adjuvant chemotherapy	4 × AC → 4 × T
		Adjuvant radiotherapy	SIB-IMRT to the whole right breast (whole-50.4Gy, high-risk area-60.2Gy)
	DFS	24 months	
	Recurrent diagnosis	March, 2018 4 o'clock of the right breast	
	Recurrent pathology	Metaplastic squamous cell carcinoma	
		Immunohisto-chemistry	ER(–), PR(–), Her-2(–), CK5/6(+), Ki-67(+,70%), EGFR(+), E-Cadherin(+)
	Treatments	Surgery	Breast modified radical mastectomy
		Adjuvant chemotherapy	3 × TP → 3 × docetaxel+capcitabine
		Adjuvant radiotherapy	IMRT to the left supraclavicular and apical axillary regions (50Gy)
	DFS	16 months (last follow-up time: August, 2019)	

(–): Negative expression; (+): Positive expression.

*4 × AC → 4 × T: four cycles chemotherapy of doxorubicin and cyclophosphamide followed by four cycles of docetaxel*.

*SIB-IMRT: simultaneous integrated boost intensity modulated radiotherapy*.

*6 × TP: Six cycles chemotherapy of docetaxel and cisplatin*.

*DFS, disease-free survival*.

After surgery, the patient received adjuvant chemotherapy with four cycles of doxorubicin and cyclophosphamide followed by four cycles of docetaxel every 3 weeks. She then underwent adjuvant radiation with simultaneous integrated boost intensity modulated radiotherapy (SIB-IMRT) to the whole right breast to a total dose of 50.4 Gy in 28 fractions and to the high-risk area for recurrence to a total dose of 60.2 Gy in 28 fractions (Supplementary Figure 1). Since the surgery, there had been no evidence of recurrence or metastasis for 24 months' follow-up.

**Figure 3:**
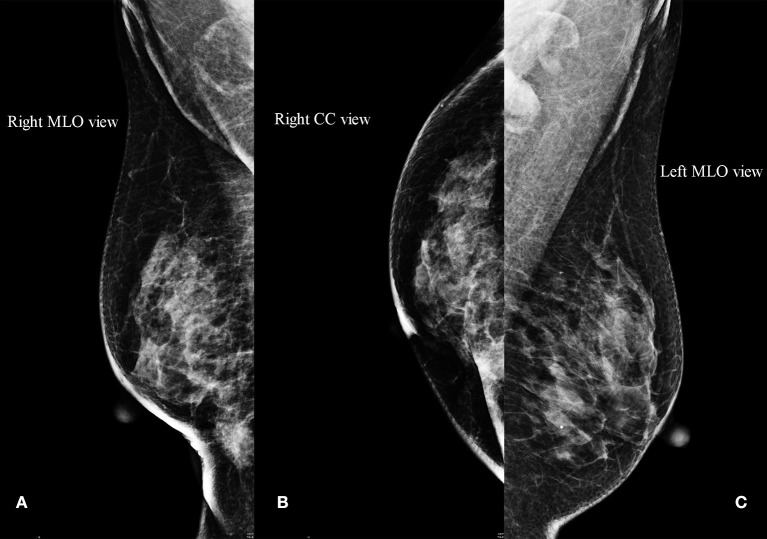
Mammogram. **(A,B)** An abnormal density shadow in the lower inner quadrant of the right breast suspected to be malignant lesions. **(C)** Enlarged left axillary lymph nodes.

**Figure 4:**
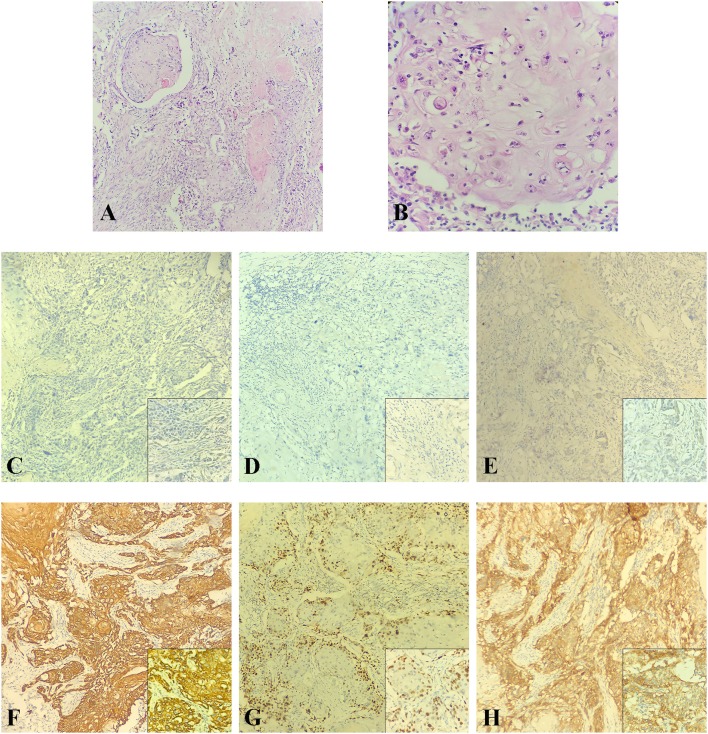
The postoperative pathological examination with HE staining and IHC staining of the recurrent tumor in 2018. **(A,B)** Histological findings with HE staining showed that the recurrent tumor was a metaplastic squamous cell carcinoma with prominent keratinization that exhibited an infiltrative growth pattern. **(A)** Original magnification (10×). **(B)** High magnification (40×). **(C–H)** IHC staining was used to detect the expressions of ER, PR, HER-2, CK5/6, Ki-67, and EGFR at original magnification (10×). **(C)** ER negative. **(D)** PR negative. **(E)** HER-2 negative. **(F)** CK5/6 positive. **(G)** Ki-67 positive (70%). **(H)** EGFR positive.

Then the patient felt a nodule in the right breast again, and the size of this nodule gradually increased. Four months later, she received an ultrasound examination, which found that there was an irregular non-homogeneous-echo mass in the lower inner quadrant of the right breast below the surgical incision and had biopsy-proven disease recurrence in the right breast which revealed SCC. A mammogram showed an abnormal density shadow in the lower inner quadrant of the right breast, which lead to suspicion of malignant lesions, and enlarged left axillary lymph nodes (Figure 3), which biopsy indicated to be invasive carcinoma. The multidisciplinary team (MDT) conference board recommended bilateral breast modified radical mastectomy on March 15, 2018. An invasive carcinoma in the right mammary gland was localized in the lower inner quadrant, measuring 1.6 × 1.5 × 1.3 cm, and 1 of the 15 left axillary lymph nodes removed was malignant. No abnormality was found in the left breast or right axillary lymph nodes. Microscopic examination of the tumors revealed metaplastic SCC with an ER-negative, PR-negative, Her-2–negative, CK5/6-positive, P53-positive 50%, EGFR-positive, and Ki-67–positive 70% phenotype detected by immunohistochemical staining (Figure 4; Table 1).

Following surgery, the case was then discussed in an MDT conference because of the change of pathological type. Adjuvant chemotherapy with six cycles of docetaxel and cisplatin was planned (TP chemotherapy). But after three cycles, the TP chemotherapy was stopped because of severe drug-related gastrointestinal adverse events. The patient then accepted docetaxel and capecitabine for another three cycles. From October 10, 2018, to November 15, 2018, she underwent adjuvant radiotherapy with intensity modulated radiotherapy (IMRT) to the left supraclavicular and apical axillary regions with a total dose of 50 Gy in 25 fractions (Supplementary Figure 2). There has been no recurrent or metastatic evidence in the 16 months' follow-up since the second surgery.

## Discussion

SCC of the breast was first reported by Troell in 1908 (6). Because SCC of the breast was a very rare type of malignancy, no large prospective randomized clinical trials have been performed to study its pathogenesis, specific radiologic characteristics, effective treatment management, and prognosis. As a metaplastic carcinoma, SCC also shows heterogeneous characteristic features. The question of the origin of metaplastic breast cancers is still unclear. Behranwala et al. proposed two theories explaining the development mechanism of SCC of the breast: (1) arising from benign breast disease and (2) arising from invasive duct carcinoma (7). However, van Deurzen et al. demonstrated that the phenotypic changes of breast cancer are the result of malignant transformation of breast cancer stem/progenitor cells (histogenesis) or specific genetic mutations taking place at early or late stages of carcinogenesis (dedifferentiation) (8). Furthermore, Avigdor et al. performed whole exome sequencing for eight patients containing both conventional *in situ* or IDC and metaplastic components, which showed that the genomic landscape of an intertwined metaplastic breast tumor may generally be the same as the non-metaplastic component, and the different histologies of these cancers may be driven mainly by epigenetic or non-coding changes (9). As for our patient, her recurrent pathological histology was a pure metaplastic SCC, which is classified as pure epithelial type according to the World Health Organization (WHO), (3) indicating that her phenotypic change was more likely the result of malignant transformation of breast cancer stem/progenitor cells.

SCC may have some common clinicopathological features, such as the larger tumor size and larger proportion of T3–4, grade III, and triple-negative (lack of expression of ER, PR, and HER2) tumors than IDC (10). It presents less frequent lymphovascular invasion, but there is no difference in occurrence of positive lymph nodes between SCC and IDC patients (10). According to previous studies, 3–4% of SCC patients develop distant metastases (7, 10). However, there are no specific imaging characteristics for the metaplastic SCC patients. The pathological criteria for the diagnosis of SCC of the breast are as follows: (a) nipple, skin, or its appendages are not the sources of the tumor; (b) over 90% of tumor areas must be squamous cells; (c) other invasive components (ductal or mesenchymal) do not exist in the whole tumor; and (d) other sites of primary SCC (PSCC) are excluded (11). The recurrent tumor in this case satisfied all of these conditions. Significantly, the pathological type of the first resected malignant tumor in the right breast was IDC, but after adjuvant chemotherapy and radiotherapy, the type of the recurrent mass changed to metaplastic SCC This indicated that pathological phenotypes of tumors could transform one type to another, especially for those with metaplastic components (6). Graziano et al. (12) reported that IDC of the breast could progress to metaplastic SCC after induction chemotherapy. Previous research revealed that long-standing breast implants and acute unilateral breast pain and enlargement might also cause secondary PSCC of the breast (13, 14). Furthermore, PSCC of the breast possibly arises from previous radiation (15). Above all, metaplastic SCC of the breast can be induced by a breast implant, chemotherapy, or radiation.

PSCC of the breast is usually a highly malignant and hormone receptor–negative tumor, which indicates that hormone-based therapy may not have an effect on these tumors. Besides, HER2-targeted therapy is not greatly effective either for negative Her-2 gene amplification. Treatment strategies of PSCC are not different from those of other triple-negative histologic types of breast tumors, which include neoadjuvant therapy, surgery, chemotherapy, hormonal therapy, and radiotherapy. Liu et al. (6) found that TP as neoadjuvant therapy may be effective for those breast PSCC patients. Several studies revealed that compared with IDC, the PSCC patients of the breast have more rapid progression, shorter overall survival, and poorer prognosis (6, 7, 10). For this patient, the second surgery was decided to perform bilateral breast modified radical mastectomy because we are not sure about the origin of the left metastatic axillary lymph node. It may be metastasized from the tumor of the right breast or from an occult cancer of the left breast. Then we used TP chemotherapy as the second adjuvant chemotherapy and adjuvant radiotherapy in the left supraclavicular and apical axillary regions. There has been no recurrent or metastatic evidence till now. Some researchers studied the landscape of somatic genetic alterations of breast PSCC patients, which demonstrated that TP53 and PI3KCA gene mutation might be caused by the activation of the Wnt and PI3K/AKT/mTOR pathway, (16) and this may provide us a new treatment strategy for breast SCC patients. Besides, epigenetic therapies may be effective treatments for metaplastic breast carcinomas (9). But the most therapeutic regimen for this rare disease still remains unclear. Therefore, knowing its pathogenesis, clinical features, and specific imaging characteristics is crucial to choose the optimal treatment for such a rare and aggressive disease.

In conclusion, this case report shows that evolution of pathology type in recurrent breast cancer after initial treatment is possible. Detailed pathologic and immunohistochemical analyses are needed for identification of this change. Surgery and adjuvant radiation and chemotherapy are appropriate treatments for recurrent PSCC of the breast.

## Data Availability Statement

All data sets generated for this study are included in the article/Supplementary Material.

## Ethics Statement

The authors of this manuscript obtained patient consent for publication of clinical data and images. The patient's details were anonymized, and the patient signed the consent form for the publication. Due to the retrospective and non-interventional nature of the study, permission by the local ethics committee was not required.

## Author Contributions

TG, ZC, JX, and YZ collected the patient's data and provided the figures. TG designed the study and finished the original manuscript. JX revised the manuscript and provided the immunohistochemical figures. YZ provided final approval for the version to be published. The final version of the manuscript was read and approved by all authors.

### Conflict of Interest

The authors declare that the research was conducted in the absence of any commercial or financial relationships that could be construed as a potential conflict of interest.
