# Supplementary material for: Change of Pathological Type to Metaplastic Squamous Cell Carcinoma of the Breast During Disease Recurrence: Case Report and Literature Review
Source: Front Oncol. 2020 Feb 25;10:32. doi: 10.3389/fonc.2020.00032 (PMC7052350; doi:10.3389/fonc.2020.00032)
Supplement: Supplementary file 1 [file Data_Sheet_1.pdf]

Supplementary Material

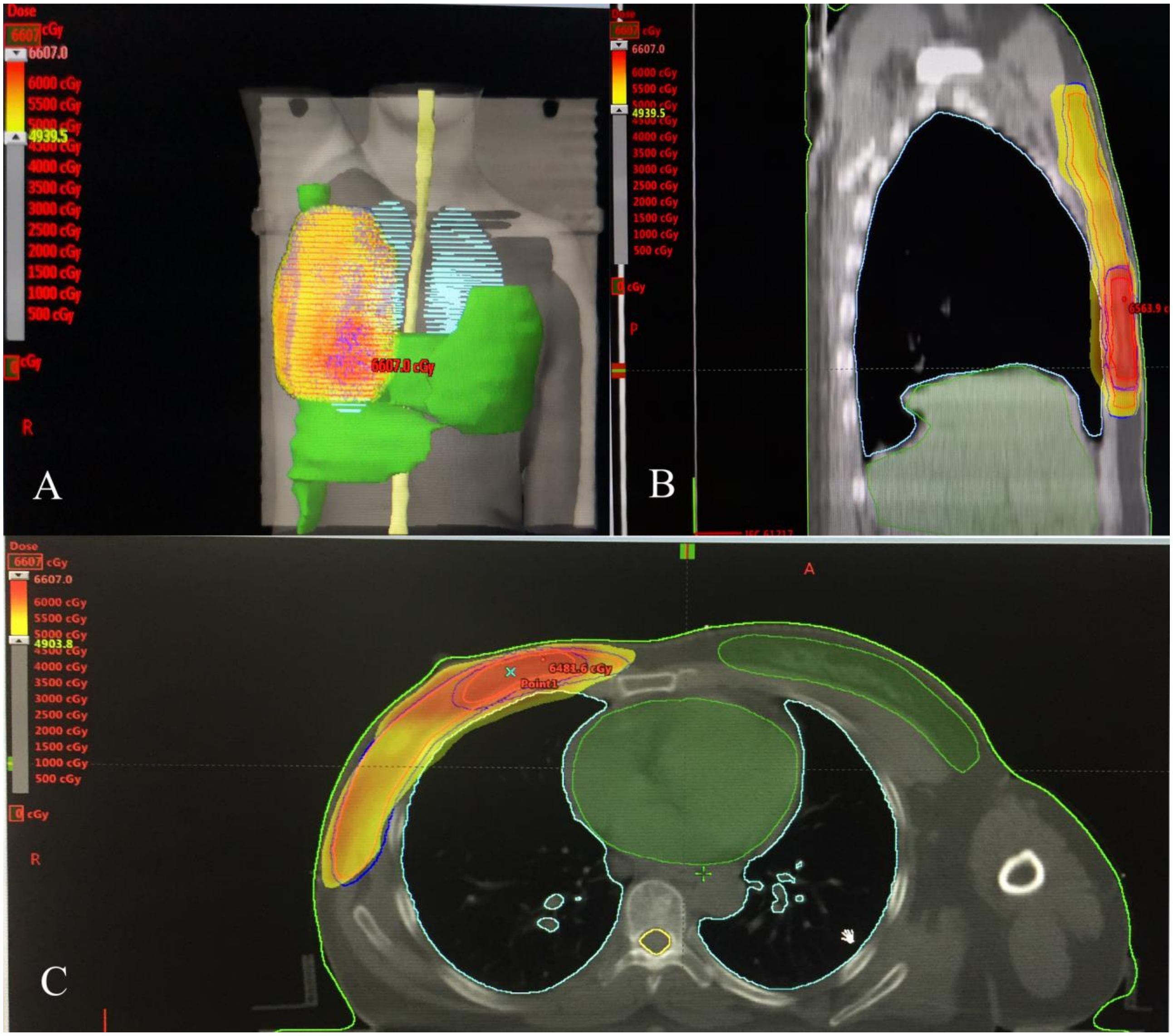

Supplementary Figure 1. Figures of the first time radiation dose distribution. SIB-IMRT was used in the whole right breast of a total dose of 50.4 Gy in 28 fractions and the higher risk for recurrence of a total dose of 60.2 Gy in 28 fractions.

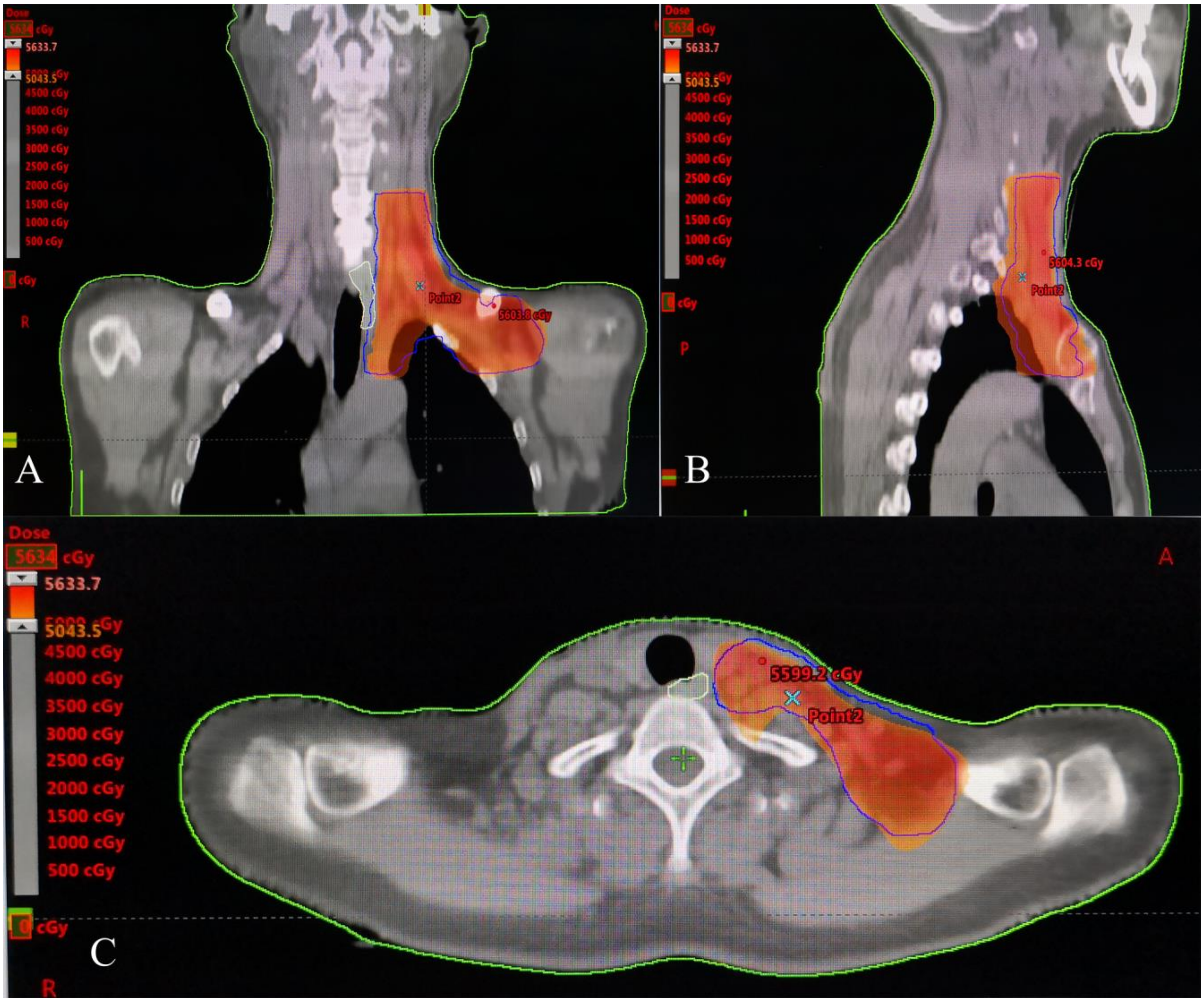

Supplementary Figure 2. Figures of the radiation dose distribution for the recurrent lesions. IMRT was used in the left supraclavicular and apical axillary regions with a total dose of 50 Gy in 25 fractions.
